# On the Use of the Bromides in Cases of Lead or Mercurial Poisoning

**Published:** 1869-01

**Authors:** 


### On the Use of the Bromides in Cases of Lead or Mercurial Poisoning.

It is well known that several years ago Natalis Guillot and Melsens recommended the iodide of potassium as the best agent for removing lead and mercury from the system. Dr. Raberteau, who has been performing some interesting experiments relative to the action of bromine and the bromides, has been led to substitute these latter for the iodides as eliminating agents of the metals in question. He was brought to the idea by observing that the bromides are very slowly removed from the system, while the iodides pass out soon after administration. The former, therefore, are disposed to accumulate in the body, and hence have a greater opportunity of dissolving the lead or mercury which may be in the tissues. In illustration of this theory, M. Raberteau cites the following interesting experiment:

He gave a dog 20 centigrammes (about 3 grains) of the acetate of lead. Saturine poisoning was produced, and continued till, at the end of several days, he administered 10 grammes (150 grains) of the bromide of potassium in two doses. The effect was, that all the symptoms of lead-intoxication soon disappeared.

M. Raberteau also adduces the case of a patient, blind, as was believed, from the effects of lead, and whose condition was greatly improved by the bromide of sodium. He prefers this salt to the bromide of potassium, on the ground that it is harmless, while the latter is poisonous.

He then refers to a case previously published, in which a man, affected with mercurial trembling, headache, and obstinate insomnia, was cured by the use of the bromide of potassium, after the iodide had failed.—*Gazette Hebdomadaire.*

